# High flow nasal cannula oxygen and non-invasive mechanical ventilation in management of COVID-19 patients with acute respiratory failure: a retrospective observational study

**DOI:** 10.1186/s43168-021-00063-0

**Published:** 2021-03-22

**Authors:** Amr Mounir Shoukri

**Affiliations:** Department of Chest Diseases, Faculty of Medicine, Ain Shams University, 43 ElMahrouky street, Heliopolis, Cairo, 11341 Egypt

**Keywords:** High flow nasal cannula oxygen, COVID-19, Non-invasive ventilation, Intensive care unit

## Abstract

**Background:**

High flow nasal cannula oxygen (HFNCO) is a relatively new technique used to deliver oxygen in respiratory failure patients. This retrospective study is aiming to assess the role and benefits of using HFNCO compared to non-invasive ventilation (NIV) in management of patients with acute hypoxemic respiratory failure associated with coronavirus disease 2019 (COVID-19).

**Results:**

A retrospective analysis of the files of 63 patients with COVID-19 and acute hypoxemic respiratory failure admitted to the intensive care unit (ICU), 37 patients received HFNCO as initial therapy, and 26 patients were primarily treated with NIV. There was no significant difference between the 2 groups in terms of baseline characteristics, laboratory tests, arterial blood gases, PaO2/FiO2 values, and vital signs. Re-assessment after 24 h of starting treatment with either HFNCO or NIV showed significant improvement (*P*<0.01) in the respiratory rate, heart rate, and oxygenation parameters. The magnitude of improvement of the vital signs and oxygenation was not significantly different between patients using HFNCO or NIV. Success rate of HFNCO was 86.4%, endotracheal intubation with invasive mechanical ventilation was required in 10.81% of patients, and mortality rate was 2.7%. Success rate of NIV was 84.6%, endotracheal intubation rate was 11.53%, and mortality rate was 3.8%. No significant difference (*P*>0.05) between the 2 groups as regards the duration of treatment, rate of endotracheal intubation with invasive mechanical ventilation, and mortality rate.

**Conclusion:**

High flow nasal cannula oxygen (HFNCO) is effective in the management of acute hypoxemic respiratory failure associated with COVID-19. Its efficacy is similar to NIV, with no difference in the duration of treatment, endotracheal intubation rate, or mortality rate.

## Background

The world is still suffering from the novel coronavirus that causes a respiratory illness named coronavirus disease 2019 (COVID-19); the clinical manifestations are diverse ranging from asymptomatic infection to acute respiratory distress syndrome that requires intensive care unit (ICU) admission with endotracheal intubation and invasive mechanical ventilation and is associated with high risk of mortality [1, 2].

About 14% of the patients infected with COVID-19 develop severe illness, and 5% of the cases are critical and usually require ICU admission with associated high risk of mortality. According to several reports, the patients admitted to ICU are often in need for oxygen with high flow or mechanical ventilation either invasive or non-invasive [3].

Non-invasive ventilation has been widely used in acute hypoxemic respiratory failure secondary to different causes, and it proved to be beneficial in COVID-19 patients admitted to ICU [3].

High flow nasal cannula oxygen (HFNCO) is a relatively new technique used in the management of acute hypoxemic respiratory failure. It delivers heated humidified oxygen through nasal prongs at high flow rates up to 60 liters/minute [4].

We aimed to retrospectively investigate the benefits of HFNCO compared to non-invasive mechanical ventilation in the management of COVID-19 patients with acute respiratory failure.

## Methods

In this retrospective observational study, we analyzed all the files of the patients admitted to ICU in Mouwasat hospital in Riyadh, Saudi Arabia, with confirmed COVID-19 associated with hypoxemic respiratory failure in the period between May 2020 and August 2020.

Consent was obtained from the patients or their relatives to use and publish their data. The study was approved by the ethics committee of the hospital.

### Inclusion criteria

Confirmed COVID-19 patients by real-time reverse transcription polymerase chain reaction (RT-PCR). All included patients had acute hypoxemic respiratory failure and received either HFNCO or NIV as initial therapy.

### Exclusion criteria

Patients who required invasive mechanical ventilation with endotracheal intubation on admission, or who did not use neither HFNCO nor NIV as initial therapy. Patients were also excluded in case of missing data necessary for analysis. Patients with no available consent to use their data for publication were also excluded.

The following data were retrieved from the patients’ files: demographics, results of baseline laboratory and arterial blood gases tests, vital signs, and baseline PaO2/FiO2 before treatment with either HFNCO or NIV. Calculated sequential organ failure assessment (SOFA) score, and Acute Physiology and Chronic Health Evaluation II (APACHE II) score at the time of admission.

Included patients used either HFNCO or NIV as initial therapy.

### HFNCO

Whenever HFNCO was used, the settings were adjusted according to published consensus and experts’ opinion [5]. The flow was set from 30–60 l/min according to the patient’s condition, and the temperature was set in the range between 31 and 37°C. The fraction of inspired oxygen (FiO2) was adjusted to keep the peripheral blood oxygen saturation (SpO2) above 93%. Close monitoring of the vital signs and arterial blood gases, and if the management with HFNCO was not successful (persistent severe symptoms, mainly dyspnea, in addition to failure in maintaining the oxygenation at the desired levels), then NIV was started if no necessary urgent endotracheal intubation, and in case of no or poor response to NIV, respiratory support was escalated with urgent endotracheal intubation and invasive mechanical ventilation according to the guidelines [6].

### NIV

Non-invasive ventilation was used according to current guidelines [7, 8]. Total face mask was the selected interface in all cases with appropriate size according to each patient. Initial inspiratory pressure was set between 8 and 10 cm H2O, and positive end expiratory pressure set at 4 cm H2O; those pressures were gradually increased and continuously adjusted according to the clinical response. The fraction of inspired oxygen (FiO2) was set and titrated based on the SpO2 aiming to maintain it above 93%. In case of no response (persistent severe symptoms, mainly dyspnea, in addition to failure in maintaining the oxygenation at the desired levels), or intolerance to NIV, we used HFNCO as a rescue if the condition did not necessitate urgent endotracheal intubation [6].

When the monitored parameters (symptoms, vital signs, hemodynamics, and SpO2) showed signs of improvement, we applied intermittent use of either HFNCO or NIV, with gradual increase in the duration of use of conventional oxygen therapy until complete weaning.

### Statistical analysis

SPSS version 20.0 for Windows (SPSS Inc., Chicago, IL, USA) was used for statistical analysis. Test results are reported as mean and standard deviations (SD) for normally distributed continuous variables. A chi-square test was performed for categorical variables. An independent sample *t* test was conducted for parametric data. *P* values of < 0.05 were considered statistically significant.

## Results

In the retrospective analysis of the files of 63 patients, 40 males (63.49%), and 26 females (36.50%), their age ranged from 36 to 81 years old, with a mean age of 66.44 ± 8.86 (Table 1). Among the included patients, 37 (58.7%) received HFNCO as initial treatment, and 26 patients (41.26%) received NIV as initial treatment. The baseline characteristics of all patients are summarized in (Table 1). There was no difference between the HFNCO group and the NIV group regarding the age and gender of the included patients; there was also no significant difference in the severity of the disease between the two groups. The associated comorbidities were diabetes mellitus, hypertension, chronic respiratory disease, chronic cardiac disease, and others like autoimmune diseases.

**Table 1:** Baseline characteristics of the included patients

	All patients (***n***=63)	HFNCO (***n***=37)	NIV (***n***=26)	***P***^**#**^
**Age (years)**	66.44 ± 8.86	67.94 ± 7.82	64.10 ± 9.81	0.065
**Gender**				
**Males (%)**	40 (63.34%)	23 (62.16%)	17 (65.38%)	
**Females (%)**	23 (36.50%)	14 (37.83%)	9 (34.61%)	
**Comorbidities**				
**Diabetes Mellitus**	21 (33.33%)	12 (32.43%)	9 (34.61%)	
**Hypertension**	19 (30.15%)	10 (27.02%)	9 (34.61%)	
**Chronic cardiac disease**	3 (4.76%)	2 (5.40%)	1 (3.84%)	
**Chronic respiratory disease**	6 (9.52%)	3 (8.10%)	3 (11.53%)	
**Others**	1 (1.58%)	1 (2.70%)	-	
**APACHE II score**	10.26 ± 3.22	9.78 ± 3.18	10.96 ± 3.15	0.07
**SOFA score**	2.88 ± 0.89	3.02 ± 0.94	2.69 ± 0.77	0.07

*HFNCO* high flow nasal cannula oxygen, *NIV* non-invasive ventilation, *APACHE* Acute Physiology and Chronic Health Evaluation, *SOFA* sequential organ failure assessment

^#^*P* for comparison between HFNCO and NIV, significance at *P*<0.05

The results of laboratory tests showed significant difference in the serum albumin and serum potassium levels between HFNCO and NIV groups, but all other laboratory tests were not not significantly different (Table 2).

**Table 2:** Laboratory tests results of the studied patients

	All patients (***n***=63)	HFNCO (***n***=37)	NIV (***n***=26)	***P***^**#**^
**Hemoglobin gm/dl**	13.60 ± 1.60	13.67 ± 1.56	13.50 ± 1.64	0.34
**White blood cells × 10**^**9**^ **/L**	5.65 ± 2.21	5.61 ± 2.19	5.7 ± 2.23	0.44
**Neutrophils %**	76.69 ± 4.46	76.32 ± 4.86	77.23 ± 3.77	0.21
**Lymphocytes count × 10**^**9**^ **/L**	0.74 ± 0.10	0.75 ± 0.09	0.71 ± 0.10	0.05
**Platelet count × 10**^**9**^ **/L**	198.34 ± 35.81	196.45 ± 34.46	201.03 ± 37.49	0.31
**Sodium mmol/L**	137.28 ± 2.23	137.13 ± 2.08	137.5 ± 2.42	0.26
**Potassium mmol/L**	3.80 ± 0.17	3.77 ± 0.15	3.86 ± 0.19	0.02*
**ALT U/L**	35.98 ± 7.84	36.37 ± 8.05	35.42 ± 7.49	0.32
**AST U/L**	38.33 ± 11.38	38.21 ± 10.15	38.5 ± 12.94	0.46
**Albumin g/dl**	3.72 ± 0.25	3.77 ± 0.24	3.63 ± 0.24	0.01*
**Serum creatinine mg/dl**	0.81 ± 0.21	0.80 ± 0.21	0.83 ± 0.24	0.31
**Blood urea nitrogen mg/dl**	18.49 ± 4.89	18.29 ± 5.20	18.76 ± 4.40	0.35
**ESR mm/h**	67.65 ± 52.40	60.43 ± 38.79	77.93 ± 65.82	0.09
**CRP mg/L**	48.53 ± 20.70	49.70 ± 21.37	46.88 ± 19.59	0.30
**Procalcitonin ng/ml**	0.06 ± 0.02	0.06 ± 0.03	0.07 ± 0.02	0.03

*HFNCO* high flow nasal cannula oxygen, *NIV* non-invasive ventilation, *ALT* alanine aminotransferase, *AST* aspartate aminotransferase, *ESR* erythrocyte sedimentation rate, *CRP* C-reactive protein

^#^*P* for comparison between HFNCO and NIV, significance at *P<0.05

The results of baseline vital signs, blood gases, and PaO2/FiO2 values were not significantly different between the 2 groups (Table 3).

**Table 3:** Vital signs and blood gas analysis of the study population before treatment

	All patients (***n***=63)	HFNCO (***n***=37)	NIV (***n***=26)	***P***^**#**^
**Heart rate (beats/minute)**	95.34 ± 6,67	95.10 ± 6.08	95.96 ± 7.40	0.36
**Respiratory rate (breaths/minute)**	28.17 ± 3.62	27.70 ± 3.11	28.88 ± 4.14	0.10
**Mean arterial blood pressure (mmHg)**	91.60 ± 4.52	91.02 ± 4.40	92.42 ± 4.55	0.11
**PH**	7.42 ± 0.03	7.43 ± 0.04	7.42 ± 0.03	0.10
**PaCO2 (mmHg)**	34.82 ± 3.77	34.67 ± 3.69	35.03 ± 3.99	0.35
**PaO2/FiO2**	190.79 ± 39.81	191.08 ± 37.83	190.38 ± 42.47	0.47

*HFNCO* high flow nasal cannula oxygen, *NIV* non-invasive ventilation, *PaCO2* arterial pressure of carbon dioxide, *PaO2* arterial pressure of oxygen, *FiO2* fraction of inspired oxygen

^#^*P* for comparison between HFNCO and NIV, significance at *P*<0.05

Vital signs, arterial blood gases, and PaO2/FiO2 measured 24 h after starting either HFNCO or NIV showed statistical significant difference compared to the baseline results (Tables 4 and 5). The use of either HFNCO or NIV was associated with significant improvement of respiratory rate, heart rat, and PaO2/FiO2 (*P*<0.01).

**Table 4:** Comparison between vital signs and arterial blood gases before and after treatment in patients using HFNCO

	HFNCO (***n***=37) Before treatment	HFNCO (***n***=37) 24 h after treatment	***P***^**#**^
**Heart rate (beats/minute)**	95.10 ± 6.08	85.29 ± 8.69	<0.01*
**Respiratory rate (breaths/minute)**	27.70 ± 3.11	21.48 ± 2.11	<0.01*
**Mean arterial blood pressure (mmHg)**	91.02 ± 4.40	89.29 ± 4.96	0.06
**PH**	7.43 ± 0.04	7.41 ± 0.03	0.02*
**PaCO2 (mmHg)**	34.67 ± 3.69	38.32 ± 4.32	<0.01*
**PaO2/FiO2**	191.08 ± 37.83	225.67 ± 44.33	<0.01*

*HFNCO* high flow nasal cannula oxygen, *NIV* non-invasive ventilation, *PaCO2* arterial pressure of carbon dioxide, *PaO2* arterial pressure of oxygen, *FiO2* fraction of inspired oxygen

Significance at *P<0.05.

**Table 5:** Comparison between vital signs and arterial blood gases before and after treatment in patients receiving NIV

	NIV (***n***=26) Before treatment	NIV (***n***=26) 24h after treatment	***P***
**Heart rate (beats/minute)**	95.96 ± 7.40	84.92 ± 6.42	<0.01*
**Respiratory rate (breaths/minute)**	28.88 ± 4.14	23.76 ± 3.58	<0.01*
**Mean arterial blood pressure (mmHg)**	92.42 ± 4.55	89.42 ± 3.91	<0.01*
**PH**	7.42 ± 0.03	7.43 ± 0.02	0.10
**PaCO2 (mmHg)**	35.03 ± 3.99	38.15 ± 3.72	<0.01*
**PaO2/FiO2**	190.38 ± 42.47	241.53 ± 49.43	<0.01*

*HFNCO* high flow nasal cannula oxygen, *NIV* non-invasive ventilation, *PaCO2* arterial pressure of carbon dioxide, *PaO2* arterial pressure of oxygen, *FiO2* fraction of inspired oxygen

Significance at *P<0.05

Comparison was done between HFNCO and NIV regarding their effects on the vital signs, arterial blood gases, and PaO2/FiO2 values, and there was no statistically significant difference between both methods, except for the respiratory rate which showed better improvement with significant decrease (*P*<0.01) in patients using HFNCO (Table 6).

**Table 6:** Comparison between HFNCO group and NIV group 24 h post treatment, as regards vital signs, arterial blood gases, and outcomes

	HFNCO (***n***=37)	NIV (***n***=26)	***P***
**Heart rate (beats/minute)**	85.29 ± 8.69	84.92 ± 6.42	0.42
**Respiratory rate (breaths/minute)**	21.48 ± 2.11	23.76 ± 3.58	<0.01*
**Mean arterial blood pressure (mmHg)**	89.29 ± 4.96	89.42 ± 3.91	0.45
**PH**	7.41 ± 0.03	7.43 ± 0.02	0.06
**PaCO2 (mmHg)**	38.32 ± 4.32	38.15 ± 3.72	0.44
**PaO2/FiO2**	225.67 ± 44.33	241.53 ± 49.43	0.09
**Duration of treatment (days)**	5.53 ± 1.11	5.86 ± 1.10	0.43
**Success rate %**	86.4%	84.61 %	0.24
**Intubation rate %**	10.81 %	11.53 %	0.34
**Mortality rate %**	2.7 %	3.8 %	0.32

*HFNCO* high flow nasal cannula oxygen, *NIV* non-invasive ventilation, *PaCO2* arterial pressure of carbon dioxide, *PaO2* arterial pressure of oxygen, *FiO2* fraction of inspired oxygen

Significance at *P<0.05

**Fig. 1:**
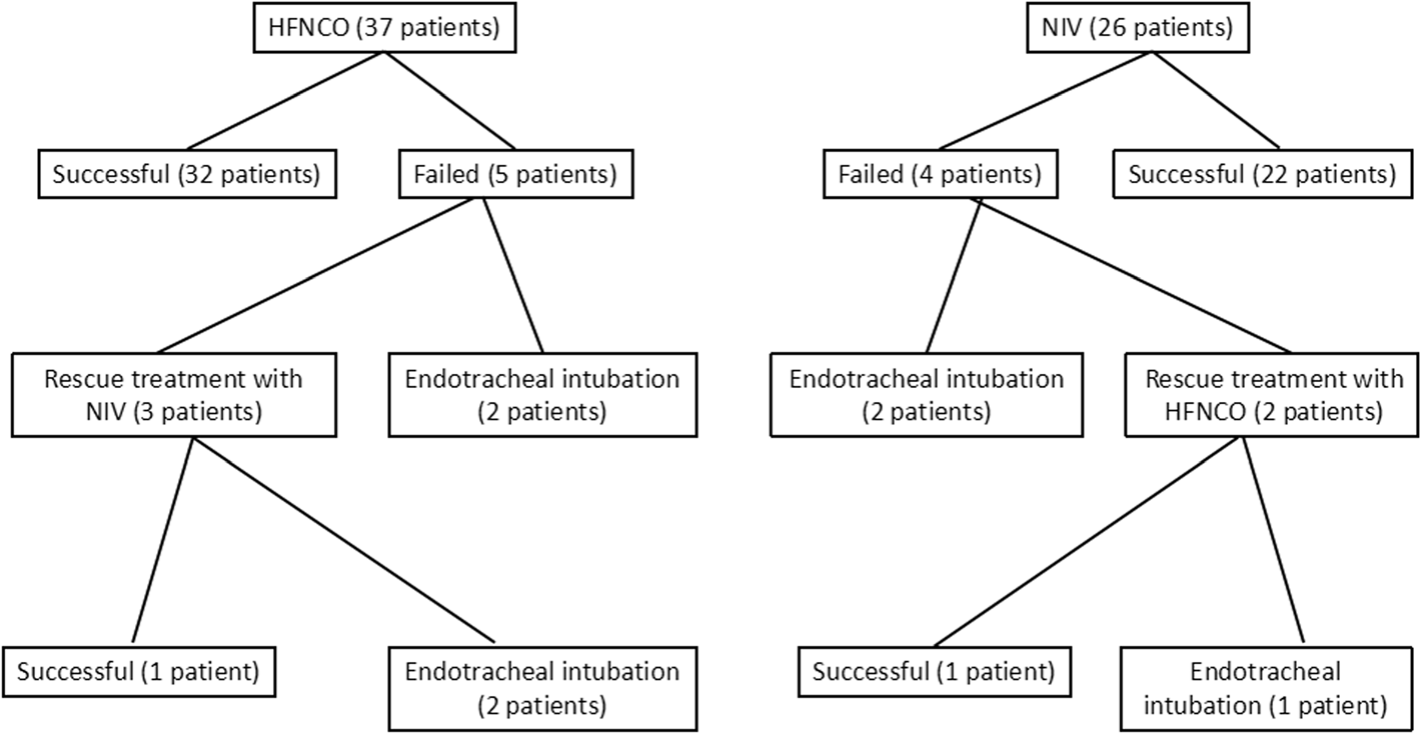
Flow chart for the outcomes of patients receiving HFNCO and NIV

Among the 37 patients who received HFNCO as the primary therapy for hypoxemia, 32 patients showed good response with no need to further escalate the respiratory support, and the mean duration of treatment with HFNCO was 5.53 ± 1.11 days (Table 6). Five patients had progressive respiratory decompensation with failed therapy with HFNCO, urgent endotracheal intubation was done for 2 patients, and 3 patients were shifted to NIV as a rescue treatment, among whom 2 patients were intubated subsequently (Fig. 1). The success rate of HFNCO as initial therapy for acute hypoxemic respiratory failure associated with COVID-19 was 86.4%, and total endotracheal intubation rate was 10.81 % (4 patients), and mortality rate was 2.7% (1 patient) (Table 6).

Among the 26 patients initially treated with NIV, 22 patients had favorable outcome, and the mean duration of treatment was 5.86 ± 1.10 days (Table 6). Two patients failed to improve with NIV and were intubated, and 2 patients did not tolerate NIV and were shifted to HFNCO as a rescue therapy which was successful in one of them, while the other patient was intubated after 2 days (Fig. 1). The success rate of NIV as initial therapy for acute hypoxemic respiratory failure associated with COVID-19 was 84.61%, total endotracheal intubation rate was 11.53 % (4 patients), and mortality rate was 3.8% (1 patient) (Table 6).

## Discussion

The World Health Organization has declared COVID-19 a global pandemic in March 2020; the primary concern is still the percentage of patients who develop severe disease with respiratory failure.

The main finding of our study is that HFNCO is successful in the management of patients with acute hypoxemic respiratory failure associated with COVID-19. When compared to non-invasive ventilation, there was no significant difference in the rate endotracheal intubation or the mortality rate, and the duration of therapy was not significantly different between the two groups. The role of humidified high flow nasal oxygen in the management of hypoxemia associated with respiratory distress is described in previous studies [9, 10].

The mean duration of treatment with HFNCO in our study was 5.53 ± 1.11 days, while the duration of treatment with NIV was 5.86 ± 1.10 days. The average rates of endotracheal intubation with invasive mechanical ventilation for patients who received HFNCO and NIV were 10.8% and 11.5% respectively. Our results are in accordance with the results of another study [11] which showed an average rate of endotracheal intubation for COVID-19 patients treated with HFNCO of 17%, and 15% for those treated with NIV; the average duration of therapy in this study [11] was 5.1 days for HFNCO and 6.8 days for NIV [11].

Two meta-analyses [12, 13] of HFNCO in hypoxemic respiratory failure patients found no added benefit to usual treatment, while another recent meta-analysis [14] found a beneficial effect of HFNCO with significant reduction of the rate of endotracheal intubation, and the benefits were comparable to NIV in terms of outcome and mortality rate [14]. In our study, HFNCO proved to be successful in managing patients with COVID-19 and acute hypoxemic respiratory failure; the rate of failure and the need to escalate the respiratory support was very low. Comparing the results of HFNCO with NIV, there was no statistical significant difference in terms of outcomes.

It has been proved that whenever intubation is indicated in patients with acute respiratory failure, it should not be delayed [15, 16]. Our choice of either HFNCO or NIV was based on the primary clinical assessment, and this did not delay endotracheal intubation and invasive mechanical ventilation for patients who required such intervention. Also, close monitoring to our patients allowed us to intervene at the right time.

In the present study, the vital signs and PaO2/FiO2 showed significant improvement 24 h following initiation of either HFNCO or NIV, there was no significant difference in the magnitude of improvement between both groups, those findings are in accordance with the findings reported in another study comparing HFNCO to NIV in hypoxemic respiratory failure patients [14], and they also reported similar improvement in patients receiving either HFNCO or NIV, with no difference in the rate of endotracheal intubation or mortality rate.

A previous study has evaluated alternating HFNCO with NIV in patients with hypoxemic respiratory failure, and they found a beneficial effects of HFNCO given in between the sessions of NIV; it helped to avoid major drops in oxygenation levels [17].

It has been previously demonstrated that NIV can improve gas exchange, decrease the rate of endotracheal intubation, and reduce the mortality in patients with respiratory failure [18]. Compared with NIV, HFNCO may have some advantages, such as greater patient comfort, easier clearance of secretions, and lower costs [17], in addition to lower incidence of different adverse events that may lead to poorer outcomes [19].

Both HFNCO and NIV are aerosol-generating procedures. Theoretically, NIV generates more aerosols than HFNCO because it generates higher pressures [20]. The transmission of infection is always a major concern when dealing with COVID-19 patients. In our study, there was no transmission of infection to any of our ICU staff, all patients were admitted in negative pressure rooms, and necessary protective equipment were available for all medical staff.

Our study has certain limitations, and the sample size is relatively small, in addition to the fact that the study is retrospective. Future prospective studies with larger samples are required to confirm our results.

## Conclusion

High flow nasal cannula oxygen (HFNCO) is effective in the management of acute hypoxemic respiratory failure associated with COVID-19. Its efficacy is similar to NIV, with no difference in the duration of treatment, endotracheal intubation rate, or mortality rate.

## Data Availability

The datasets generated during and/or analyzed during the current study are available from the corresponding author on reasonable request.

## Abbreviations

APACHE: Acute Physiology and Chronic Health Evaluation
COVID-19: Coronavirus disease 2019
FiO2: Fraction of inspired oxygen
HFNCO: High flow nasal cannula oxygen
ICU: Intensive care unit
NIV: Non-invasive ventilation
RT-PCR: Real-time reverse transcription polymerase chain reaction
SOFA: Sequential organ failure assessment
SpO2: Peripheral blood oxygen saturation
